# Vitamin D status of children with severe early childhood caries: a case–control study

**DOI:** 10.1186/1471-2431-13-174

**Published:** 2013-10-25

**Authors:** Robert J Schroth, Jeremy A Levi, Elizabeth A Sellers, James Friel, Eleonore Kliewer, Michael EK Moffatt

**Affiliations:** 1 The University of Manitoba, Winnipeg, Canada; 2 The Manitoba Institute of Child Health, Winnipeg, Canada; 3 Winnipeg Regional Health Authority, Winnipeg, Canada; 4 Department of Preventive Dental Science, Faculty of Dentistry, Department of Pediatrics & Child Health, Faculty of Medicine, University of Manitoba, 507 – 715 McDermot Avenue, Winnipeg MB R3E 3P4, Canada

**Keywords:** Early childhood caries, Vitamin D, Nutritional status, Calcium, Parathyroid hormone, Preschool children

## Abstract

**Background:**

Severe Early Childhood Caries (S-ECC) affects the health and well-being of young children. There is limited research in this area, though evidence suggests that children with S-ECC are at an increased risk of malnutrition. The purpose of this study was to determine the association between vitamin D (25(OH)D) levels and S-ECC.

**Methods:**

This case–control study was conducted from 2009 to 2011 in the city of Winnipeg, Manitoba, Canada. 144 preschool children with S-ECC were recruited from a local health centre on the day of their slated dental surgery under general anesthetic. 122 caries-free controls were recruited from the community. Children underwent a blood draw for vitamin D (25(OH)D), calcium, parathyroid hormone, and albumin levels. Parents completed an interviewed questionnaire assessing the child’s nutritional habits, oral health, and family demographics. Analyses included descriptive and bivariate statistics as well as multiple and logistic regression. A p value ≤ 0.05 was significant.

**Results:**

The mean age of participants was 40.8 ± 14.1 months. Children with S-ECC had significantly lower mean 25(OH)D (68.9 ± 28.0 nmol/L vs. 82.9 ± 31.1, p < 0.001), calcium (p < 0.001), and albumin (p < 0.001) levels, and significantly higher parathyroid hormone (p < 0.001) levels than those caries-free. Children with S-ECC were significantly more likely to have vitamin D levels below recognized thresholds for optimal and adequate status (i.e. <75 and <50 nmol/L, respectively). Multiple regression analysis revealed that S-ECC, infrequent milk consumption, and winter season were significantly associated with lower 25(OH)D concentrations. Low 25(OH)D levels, low household income, and poorer ratings of the child’s general health were significantly associated with S-ECC on logistic regression.

**Conclusion:**

Children with S-ECC appear to have relatively poor nutritional health compared to caries-free controls, and were significantly more likely to have low vitamin D, calcium, and albumin concentrations and elevated PTH levels.

## Background

Early Childhood Caries (ECC) is the most common chronic disease of childhood and is defined as any decay in the primary dentition of children < 72 months of age [1,2]. Some children develop a rampant subtype of ECC termed Severe Early Childhood Caries (S-ECC), a condition known to affect health and well-being [3]. The extent of decay that they experience generally warrants rehabilitative dental surgery under general anesthesia (GA). Unfortunately, dental surgery is the most common day surgical procedure at most Canadian pediatric hospitals [4]. While dental surgery targets the visible signs of the disease, our understanding of the systemic influence of S-ECC on overall health is limited.

Quality of life is reduced among those suffering from S-ECC [3,5,6]. This can include pain, disturbed sleep and behavioural changes [6-8]. Children with severe decay can also have altered eating habits and preferences [5,9]. Therefore, rampant caries can influence nutritional health. A few reports reveal that some may be suffering from a degree of malnutrition, specifically anaemia and low iron concentrations, and have altered growth patterns impacting height and body mass index [10-13]. It is plausible that those with S-ECC are also deficient in important vitamins and nutrients, including vitamin D [14].

Vitamin D regulates calcium levels and plays a key role in craniofacial development and the maintenance of good oral health. There are two main sources of obtaining vitamin D: endogenous synthesis and exogenous attainment from diet and supplementation [15,16]. It has a critical role in enamel, dentin, and oral bone formation as ameloblasts and odontoblasts are target cells for 1,25-dihydroxyvitamin D, the active form of vitamin D [17]. Deficiency in vitamin D during periods of tooth development may also result in developmental defects [17] including enamel hypoplasia, a significant risk factor for S-ECC. Vitamin D is associated with the two main oral diseases, caries and periodontal disease [14,18-23]. In general, higher serum levels of 25-hydroxyvitamin D (25(OH)D) are associated with improved oral health outcomes [14,20-22]. Vitamin D also has an immunological role as it can induce the production of antimicrobial peptides such as cathelicidin and certain defensins, which protect us from oral pathogens [22,24].

The purpose of this study was to determine the association between serum concentrations of 25(OH)D and S-ECC in preschool children.

## Methods

A cross-sectional case–control study was undertaken to test the hypothesis that children with S-ECC have lower serum 25(OH)D, calcium, albumin, and higher parathyroid hormone (PTH) levels than caries-free controls. Differences in ferritin and haemoglobin between these groups has previously been reported in this sample [12]. This study was approved by the University of Manitoba’s Health Research Ethics Board, the Misericordia Health Centre (MHC), and the Health Sciences Centre (HSC), Winnipeg, Canada. All parents provided written informed consent at recruitment, and a small honorarium was provided.

From October 2009 to August 2011, otherwise-healthy children with S-ECC were recruited from the MHC in Winnipeg, Canada (49° 53′ North) on the day of their dental surgery. Since the case definition for S-ECC is age specific, participants needed to be ≤ 71 months of age [25]. Age-matched caries-free controls were recruited from the community by advertisement and underwent a dental screening by a study team member (RJS). Children were assessed using the dmft index (a cumulative score of decayed, missing, filled primary teeth). Those having a dmft score of 0 were considered caries-free.

Caregivers completed a questionnaire administered by staff which collected information about the child and caregiver, dietary intakes, use of supplements, sun exposure and skin pigmentation, oral hygiene behaviours, and socioeconomic factors including household income, parental education level, and receipt of government assistance [12]. This instrument was based upon a previously piloted questionnaire [14].

Venipunctures for children with S-ECC were drawn by the attending anesthetist during surgery while blood samples from controls were obtained by a research nurse at the Manitoba Institute of Child Health following the application of a topical anaesthetic (EMLA) to the anticubital fossa. Serum analysis for calcium, PTH, and albumin was performed by the Department of Biochemistry and Genetics Laboratory at HSC. In cases where the serum albumin levels were below the appropriate thresholds for a child’s age, the corrected calcium level was used in place of the standard calcium values. Diagnostic Services of Manitoba laboratory reference ranges were adopted for calcium (2.1-2.6 mmol/L), albumin (35–47 g/L for those < 48 months and 33–39 g/L for those ≥ 48 months), and PTH (7–50 ng/L). Assays for 25(OH)D, the main circulating form of vitamin D, were conducted by the Hospitals in Common Laboratory (HICL) at Mount Sinai Hospital in Toronto, Canada using Chemiluminescence Immunoassay. The key thresholds used to quantify 25(OH)D levels within this study were ≥ 75 nmol/L (optimal based on HICL and HSC), ≥ 50 nmol/L (adequate based on Institute of Medicine (IOM)), and < 35 nmol/L (common threshold used to denote deficiency) [14,16,26-28].

A minimum sample of 120 children in each group was expected to provide 80% power to detect a one-tailed difference in 25(OH)D levels between the groups at α = 0.05. Lab and questionnaire data were entered into an Excel (Microsoft Office) spreadsheet and analyzed using Number Cruncher Statistical Software (NCSS) version 7.0 (Kaysville, Utah). Analysis included descriptive statistics (frequencies, means ± Standard Deviations (SD)), Chi-square analysis, and t-tests. Unadjusted odds ratios (OR) and 95% confidence intervals (CI) were also calculated. Multiple regression analysis was performed for mean 25(OH)D including independent variables significantly associated with vitamin D levels on bivariate analysis or known to influence vitamin D status. Logistic regression for S-ECC including variables associated at the bivariate level was also performed. In both models, some variables were excluded when there was evidence of multi-colinearity. A p value ≤ 0.05 was significant.

## Results

A total of 266 children (51.1% male) participated; 144 children with S-ECC and 122 caries-free. For various reasons, blood samples were only collected for 97.9% (n = 141) of children with S-ECC and 99.2% (n = 121) of controls. The mean age was 40.8 ± 14.1 months. The groups were well matched for age and sex as there were no significant differences (p = 0.14 and p = 0.37, respectively). Characteristics of participants and their parent or caregiver appear in Table 1. As S-ECC is influenced by the social determinants of health, there were differences in parental education levels (p < 0.001) and household income (p < 0.001) between groups, with lower levels of both education and household income in the S-ECC group. Fewer children in the S-ECC group were reported as having good or very good oral health compared to controls (19.4% vs. 95.9%, p < 0.001). Significantly more children with S-ECC had a first visit to the dentist for a dental problem (e.g. pain or caries) than controls (40.7% vs. 5.4%, p < 0.001). Additionally, fewer caregivers of children with S-ECC indicated that their child’s overall health was very good compared to those whose children were caries-free (58.3% vs. 82.8%, p < 0.001).

**Table 1:** Association between caries status and child and caregiver characteristics

**Variable**	**Overall value**	**Caries status**				**P value**
		**S-ECC**		**Caries-free**		
		**N (%)**	**95% CI**	**N (%)**	**95% CI**	
**Child**						
Child’s age (Months)^†^	40.8 ± 14.1	42.0 ± 11.9	/	39.4 ± 16.3	/	0.14
Sex^*^						
Male	136	70 (51.5)	43.1,59.9	66 (48.5)	40.1,56.9	0.37
Female	130	74 (56.9)	48.4,65.4	56 (43.1)	34.6,51.6	
Height (cm)^†^	98.4 ± 9.7	99.3 ± 8.1	/	97.3 ± 11.1	/	0.10
Weight (kg)^†^	16.4 ± 3.7	17.0 ± 3.3	/	15.8 ± 4.0	/	0.01
Skin colour^*^						
Dark/Mid-colour	116	76 (65.6)	56.9,74.2	40 (34.5)	25.8,43.1	0.001
Light	150	68 (45.3)	37.4,53.3	82 (54.7)	46.7,62.6	
Multivitamin Use^*^						
Yes	143	76 (53.1)	45.0,61.3	67 (46.9)	38.7,55.0	0.73
No	123	68 (55.3)	46.5,64.1	55 (44.7)	35.9,53.5	
Age started cleaning mouth (Months) ^†^	12.9 ± 8.6	14.5 ± 9.5	/	10.9 ± 7.1	/	<0.001
Mean age at first dental visit (Months) ^†^	26.2 ± 11.8	27.0 ± 11.9	/	24.7 ± 11.7	/	0.17
Breast-fed^*^						
Yes	204	93 (45.6)	38.8,52.4	111 (54.4)	48.0,61.0	<0.001
No	61	50 (82.0)	72.3,91.6	11 (18.0)	8.4,27.7	
Bottle-fed^*^						
Yes	194	114 (58.8)	51.8,65.7	80 (41.2)	34.3,48.2	0.01
No	71	29 (40.8)	29.4,52.3	42 (59.2)	47.7,70.6	
**Parent/Caregiver**						
Caregiver status						
Mother	247	132 (53.4)	47.2,59.7	115 (46.6)	40.0,53.0	0.62
Father	15	9 (60.0)	35.2,84.8	6 (40.0)	15.2,64.8	
Other	4	3 (75.0)	32.6,117.4	1 (25.0)	-17.4, 67.4	
Graduated high school^*^						<0.001
Grade 12 or higher	197	86 (43.7)	36.7,50.6	111 (56.3)	49.4,63.3	
< Grade 12	66	55 (83.3)	74.0,92.0	11 (16.7)	7.7,25.7	
Afford dental care^*^						
Yes	222	123 (55.4)	48.9,61.9	99 (44.6)	38.1,51.1	0.28
No	41	19 (46.3)	331.1,61.6	22 (53.7)	38.0,69.0	
Receives social assistance^*^						<0.001
Yes	103	81 (78.6)	70.7,86.6	22 (21.4)	13.4,29.3	
No	158	61 (38.6)	31.0,46.2	97 (61.4)	53.8,69.0	
Yearly household income^*^						<0.001
<$28 000	119	85 (71.4)	63.3,79.5	34 (28.6)	20.5,36.7	
>$28 000	134	47 (35.1)	27.0,43.0	87 (64.9)	56.8,73.0	

S-ECC = Severe Early Childhood Caries.

†t-test, *chi-square.

95% CI = 95% Confidence Intervals.

One child had a mean 25(OH)D level above four SD from the mean and was excluded from subsequent analyses. The mean 25(OH)D concentration for the entire sample was 75.4 ± 30.2 nmol/L. A total of 136 children (52.1%) had levels < 75 nmol/L, indicating suboptimal vitamin D while 43 children (16.5%) had inadequate vitamin D (< 50 nmol/L). Additionally, 16 (6.1%) had deficient vitamin D concentrations (< 35 nmol/L).

More frequent intake of foods containing or fortified with vitamin D (e.g. liver, eggs, fish, fortified orange juice) were not significantly associated with higher mean 25(OH)D levels (data not shown). The exception were regular milk drinkers (≥ 5 servings weekly), who had significantly higher mean vitamin D levels than non-regular milk drinkers (76.6 ± 30.3 nmol/L vs. 63.2 ± 27.0, p = 0.042). Those presently taking vitamin D drops also had higher 25(OH)D levels (92.2 ± 34.6 nmol/L vs. 72.2 ± 28.2, p < 0.001).

There were no apparent differences in the intake of foods containing vitamin D between the S-ECC and caries-free groups, including the frequency of milk consumption (data not shown). However, significantly more children receiving vitamin D drops belonged to the caries-free group (14.0% S-ECC (n = 6) vs. 86.0% caries-free (n = 37), p < 0.001). There was also no significant difference in multivitamin usage between the groups (76 with S-ECC vs. 67 caries-free, p = 0.73). Significantly more children in the S-ECC group were bottle-fed compared to controls (p = 0.01) and bottle-fed to a later age (19.7 ± 8.7 months vs. 16.4 ± 7.7, p = 0.02). Meanwhile, fewer children with S-ECC were breastfed compared to the caries-free group (p < 0.001). There was no difference in the frequency of daily “between-meal” snacking between the groups (93.8% S-ECC vs. 96.7% caries-free, p = 0.39, Fisher’s Exact Test).

There was no significant difference between the two groups with respect to premature birth (p = 0.88), whether mothers took vitamin D supplements during pregnancy (p = 0.92), or maternal milk intake during pregnancy (p = 0.56) (data not shown).

Mean 25(OH)D levels were significantly lower among children with S-ECC than caries-free controls (68.9 ± 28.0 nmol/L vs. 82.9 ± 31.1, p < 0.001) (Table 2). Even after stratifying by season and only analyzing data collected during the winter (October-April) to control for endogenous production, a statistically significant difference remained (63.5 ± 27.7 nmol/L vs. 79.4 ± 26.7, p < 0.001).

**Table 2:** 25(OH)D, Calcium, Albumin, and PTH status by S-ECC and caries-free group

**Variable**	**Overall value**	**Caries status**				**P value**
		**S-ECC**		**Caries-Free**		
		**N (%)**	**95% CI**	**N (%)**	**95% CI**	
**25(OH)D status**						
Mean (nmol/L) ^†^	75.4 ± 30.2	68.9 ± 27.9	/	82.9 ± 31.1	/	**<**0.001
Optimal* (≥ 75 nmol/L)						
Yes	125	56 (44.8)	36.1,53.5	69 (55.2)	46.0,64.0	0.006
No	136	84 (61.8)	53.6,69.9	52 (38.2)	30.1,46.4	
Adequate* (≥ 50 nmol/L)						
Yes	218	111 (50.9)	44.3,57.6	107 (49.1)	42.4,55.7	0.05
No	43	29 (67.4)	53.4,81.4	14 (32.6)	18.6,46.6	
Deficient* (< 35 nmol/L)						
Yes	16	12 (75.0)	53.8,96.2	4 (25.0)	3.8,46.2	0.12^a^
No	245	128 (52.2)	46.0,58.5	117 (47.8)	41.5,54.0	
**Calcium status**						
Mean (mmol/L) ^†^	2.3 ± 0.1	2.2 ± 0.1	/	2.4 ± 0.1	/	<0.001
Low calcium*						
Yes	10	10 (100.0)	100,100	0 (0.0)	0,0	0.002^a^
No	248	130 (52.4)	46.2,58.6	118 (47.6)	41.4,53.8	
Low calcium (Corrected)*						
Yes	6	6 (100)	100,100	0 (0.0)	0,0	
No	252	134 (53.2)	47.0,59.3	118 (46.8)	41.0,53.0	0.03^a^
**PTH status**						
Mean (ng/L) ^†^	47.3 ± 22.0	59.1 ± 21.8	/	32.9 ± 10.8	/	<0.001
Elevated PTH*						
Yes	92	85 (92.4)	87.0,97.8	7 (7.6)	2.2,13.0	<0.001
No	162	55 (34.0)	26.7,41.2	107 (66.0)	58.7,73.3	
**Albumin status**						
Mean (g/L) ^†^	38.4 ± 3.7	36.8 ± 3.1	/	40.3 ± 3.5	/	<0.001
Low albumin*						
Yes	33	26 (78.8)	64.8,92.7	7 (21.2)	7.3,35.2	0.002
No	225	114 (50.7)	44.1,57.2	111 (49.3)	43.0,56.0	

S-ECC = Severe Early Childhood Caries.

†t-test, *chi-square, ^a^Fisher’s Exact Test.

95% CI = 95% Confidence Intervals.

The 25(OH)D distribution based on caries-status appears in Table 2. Those in the S-ECC group were found to have relatively poor vitamin D status compared to caries-free children. Significantly more children with S-ECC had suboptimal 25(OH)D concentrations (< 75 nmol/L) compared to their caries-free peers (p = 0.006) (Table 2). In fact, children with 25(OH)D levels below this threshold were twice as likely to have S-ECC. This relationship was also present when the IOM threshold for adequacy (50 nmol/L) was applied (p = 0.05, OR = 0.5). When the “deficient” threshold (< 35 nmol/L) was applied, this relationship failed to reach significance (p = 0.12 (Fisher’s Exact Test), OR = 2.7).

There was no association between the education level of the primary caregiver and the child’s vitamin D status with respect to both the mean levels and the proportion with levels ≥ 75 nmol/L (p = 0.74 and p = 0.35, respectively). However, higher yearly household incomes were associated with higher mean 25(OH)D levels and having concentrations ≥ 75 nmol/L (p = 0.002 and p = 0.002, respectively).

Differences in mean calcium, albumin, and PTH concentrations between the groups are also reported in Table 2. Children with S-ECC had significantly lower mean calcium (p < 0.001) and mean albumin (p < 0.001) levels as well as higher mean PTH concentrations (p < 0.001) than controls. Despite small cell sizes, it should be noted that all of the children with low calcium concentrations belonged to the S-ECC group. Children with S-ECC were 23.6 times more likely to have elevated PTH levels compared to controls and 3.6 times more likely to have abnormally low albumin concentrations (Table 2).

Multiple regression for 25(OH)D concentrations revealed that levels were significantly and independently associated with S-ECC, regular milk consumption, and season of assessment but not household income and or the use of vitamin D drops (Table 3). S-ECC, infrequent milk intake, and winter season were associated with lower 25(OH)D levels, but not household income or vitamin D drop use. Logistic regression for caries status was also performed, revealing that lower household income, poorer ratings of children’s general health, and lower vitamin D levels were significantly associated with S-ECC (Table 4).

**Table 3:** Multiple regression for mean vitamin D level

**Variable**	**Unstandardized regression coefficient (b)**	**± 95% confidence interval**	**P value**
Intercept	71.01	/	/
S-ECC (Reference = Yes)	5.33	1.24, 9.42	0.01
Regular milk drinker (Reference = Yes)	-6.60	-13.16, -0.03	0.05
Season (Reference = Winter)	5.22	1.37, 9.06	0.008
Vitamin D drop user (Reference = Yes)	-2.17	2.01, -6.13	0.28
Yearly income (Reference = < 28 k)	2.73	2.06, -1.32	0.19

S-ECC = Severe Early Childhood Caries.

Adjusted R^2^ = 8.37%.

**Table 4:** Logistic regression for S-ECC

**Variable**	**Adjusted odds ratio**	**± 95% confidence interval for odds ratio**	**P value**
Age started cleaning teeth	0.97	0.94, 1.01	0.12
Bottle fed (Reference = Yes)	0.73	0.37, 1.46	0.37
General health (Reference = Very Good)	3.04	1.50, 6.15	0.002
Mean vitamin D	1.01	1.00, 1.02	0.04
Vitamin D drop usage (Reference = Yes)	1.51	0.81, 2.82	0.19
Yearly income (Reference = < 28 k)	0.30	0.16, 0.55	<0.001

S-ECC = Severe Early Childhood Caries.

## Discussion

The purpose of this study was to determine whether children with S-ECC have different nutritional profiles than their caries-free peers, specifically vitamin D, calcium and albumin status. While historical evidence suggests that vitamin D supplementation can prevent caries onset and progression, much of this research has been overlooked [18,29,30]. In a pilot study published in 2012, we were the first to specifically report differences in actual serum 25(OH)D levels between children with and without severe decay [14]. The present study involved a substantially larger sample, and reinforces the observed association between 25(OH)D levels and S-ECC.

Much of the early research in this field was conducted by May Mellanby, who identified an association between vitamin D supplementation and reduced caries-risk [18,31]. Recently, a meta-analysis reported that vitamin D supplementation in childhood can help prevent caries [30], and it has also been suggested that concentrations of 25(OH)D between 75–100 nmol/L may reduce the risk for caries [22]. Our study supports these findings, as caries-free children were twice as likely to have optimal 25(OH)D concentrations (≥ 75 nmol/L) and those with S-ECC were at nearly three times the odds of having deficient levels (< 35 nmol/L).

The mean 25(OH)D level in our sample was adequate, mirroring our pilot study findings [14] and those of the Canadian Health Measures Survey [32]. This may be attributed to regular milk intake, which was common in both groups. Regular milk drinkers had better vitamin D concentrations, a finding supported by a large-scale clinical study observing an association between frequent milk consumption and increased 25(OH)D [33].

Regression techniques were employed to determine whether the association between caries and vitamin D status remained after controlling for confounders. Even after controlling for seasonal influence on endogenous synthesis, low household income, and infrequent consumption of vitamin D drops and milk, the significant association remained. Similarly, the association between 25(OH)D concentrations and S-ECC was significant after logistic regression while controlling for factors such as general health status, the age when teeth cleaning was first started, and household finances. As there was a strong relationship between education and income, education was not included in the model to avoid multi-colinearity.

Our study also reports that those with S-ECC had significantly lower calcium and elevated PTH levels, as observed in our pilot study [14]. These metabolites were not, however, included in the regression models as they were strongly correlated with 25(OH)D levels. This multi-colinearity is expected, as these variables are physiologically interrelated.

Children with S-ECC were found to have significantly lower albumin levels than caries-free controls. Our finding that 18.6% in the S-ECC group had low albumin is comparable to the 15% reported in another Canadian sample. Albumin is a serum protein that can be used as an additional indicator of overall nutritional status and malnutrition [34]. A deficiency in this protein in conjunction with undesirable vitamin D, PTH, and calcium levels may suggest that children with S-ECC have nutritional deficiencies. We have recently reported that children with S-ECC from this same study group were more likely to have low ferritin and hemoglobin levels along with iron deficiency anaemia [12]. Others have also reported that rampant caries can negatively impact nutritional health status and well-being [3,10,13,35]. Therefore, health professionals should be aware of the potential nutritional deficiencies in children suffering from extensive dental caries.

It is important to consider how S-ECC and poor nutritional status are connected. Vitamin D and calcium disturbances during tooth development may result in dentin and enamel defects, which can increase the risk for caries. However, children with S-ECC may experience ongoing pain which may alter their eating habits. This can improve after dental surgery under GA [3,9]. Avoidance of food because of severe dental problems may, in turn, contribute to the nutritional deficiencies identified in this sample [12].

This case–control study has some limitations. While we did not assess caries rates, all children with S-ECC had multiple cavitated caries lesions necessitating surgery. The cross-sectional design did not allow us to distinguish between cause and effect. Additionally, children in our study were matched by age and sex, but we were unable to match by household economics and caregiver education levels. The majority of S-ECC children came from lower-income households. Identifying caries-free controls living in these same communities proved challenging. Naturally, some factors are difficult to control for as they are critical to explaining why children are at risk for caries (e.g. household income, parental education, etc.). Fortunately, we controlled for household income in the regression models. Additionally, the caregiver questionnaire involved retrospective questions on prenatal diet and the child’s first 12 months of life which may have introduced recall bias. In retrospect, a comprehensive food frequency assessment would have been a useful addition to the study. Despite these limitations, the large sample size provided sufficient statistical power, allowing greater confidence in our findings.

## Conclusion

Based on the findings of this study, we conclude that:

• Children with S-ECC appear to be at significantly greater odds of having low vitamin D status compared to their caries-free controls.

• Children with S-ECC are likely malnourished, as they displayed significantly lower levels of calcium and serum albumin as well as higher levels of PTH compared to the control group.

This study suggests a clear relationship between vitamin D levels and the caries status of preschool children. As a result of these findings, it may be advantageous for primary care providers (including dentists and physicians) to consider serum 25(OH)D status when assessing the child’s overall health. Specifically, recommending vitamin D supplementation for children at risk of dental caries may result in a decrease in the overall prevalence of S-ECC and, ultimately, reduce the burden on pediatric day surgery centres.

## Abbreviations

CI: Confidence intervals; dmft: decayed, missing, filled teeth; ECC: Early childhood caries; GA: General anesthesia; HSC: Health sciences centre; IOM: Institute of medicine; MHC: Misericordia health centre; OR: Odds ratio; PTH: Parathyroid hormone; SD: Standard deviation; S-ECC: Severe early childhood caries.

## Competing interests

The authors declare that no competing interests (financial or personal) exist with regards to this manuscript.

## Authors’ contributions

RS: Conception and design, acquisition of data, analysis and interpretation of data, drafting of article, revising article critically for important intellectual content, and final approval of version to be published. JL: Acquisition of data, analysis and interpretation of data, drafting of article, revising article critically for important intellectual content, and final approval of version to be published. ES: Analysis and interpretation of data, revising article critically for important intellectual content, and final approval of version to be published. JF: Analysis and interpretation of data, revising article critically for important intellectual content, and final approval of version to be published. EK: Acquisition of data, revising article critically for important intellectual content, and final approval of version to be published. MM: Conception and design, analysis and interpretation of data, revising article critically for important intellectual content, and final approval of version to be published. All authors read and approved the final manuscript.

## Pre-publication history

The pre-publication history for this paper can be accessed here:

http://www.biomedcentral.com/1471-2431/13/174/prepub
